# Basic research into Women’s Health should be a priority for all

**DOI:** 10.1371/journal.pbio.3003990

**Published:** 2026-08-28

**Authors:** Joanna Clarke, on behalf of the PLOS Biology Staff Editors

**Affiliations:** Public Library of Science, San Francisco, California, United States of America and Cambridge, United Kingdom

## Abstract

Despite recent progress in clinical research into Women’s Health, basic research is still lagging behind. This Editorial calls for a renewed focus on basic physiology research into both sexes to close the gap.

## Teaser

Despite recent progress in clinical research into Women’s Health, basic research is still lagging behind. Until we understand the fundamental physiology of women across the life span, we cannot expect to bring about meaningful change.

How do pregnancy and menopause affect the brain? What happens to the placenta during pre-eclampsia? How do hormone changes throughout a woman’s lifespan affect whole-body metabolism? These seem like basic physiology questions, but we know surprisingly little about them. And this lack of knowledge has serious repercussions for around half of the global population, many of whom will be affected by these body changes and conditions at some point during their lives.

Yet, the gaps in our knowledge are not restricted to female-only health concerns. Life-threatening conditions such as cardiac arrest and stroke present differently in men and women [1], but whether there are underlying physiological differences behind these phenomena has not yet been fully explored. We also do not fully understand how the immune system functions differently in women compared with men and how this affects susceptibility to infectious and non-infectious diseases. Or what happens to these differences as we age and how this affects healthcare outcomes. A better understanding of the fundamental physiological differences between males and females is likely to have far-reaching consequences and have the potential to improve healthcare and well-being for everyone.

Research into sex differences in physiology has suffered from the predominant use of males in animal studies [1], the idea being that males were easier to study because they did not have messy hormone cycles and could not become pregnant (although this ignored the fact that males do have hormone cycles, which are also chronically understudied). This is similar to the historical preference for using male participants in clinical trials. However, the assumption that, with the exception of reproductive biology, what happens in males would be the same in females has led to a constrained view of physiology. To more fully understand human biology in a way that will be beneficial for all, we need to embrace the messiness of complex hormone cycles so that we can investigate how they affect the body beyond the reproductive system. And we need to be mindful of the important role of the X chromosome in disease risk and immune functions and how this affects everyone (we all have at least one X chromosome per cell).

Looking more specifically at what is normally considered ‘Women’s Health’, research in this area has historically been underfunded. In 2020, only 1% of global research and development funding was allocated to non-cancer-related Women’s Health [2] and, according to the World Economic Forum, only 6% of private healthcare capital is spent on conditions that predominantly or solely affect women [3]. Moreover, a recent analysis showed that funding from the US National Institutes of Health between 2022 and 2024 for male-predominant conditions was 5.2 times greater per year lived with disability than for female-predominant conditions [4]. Attempts to redress this imbalance have begun, with Women’s Health research initiatives and funding calls [5,6] pushing the topic more into the limelight, but much of the focus currently lies on improving clinical outcomes [7] rather than on understanding the underlying biology. Improvements in diagnostics, monitoring technology, and treatments are desperately needed for many health conditions that predominantly or solely affect women and those born with female reproductive organs (yes please to a blood test for cervical cancer), but the focus on healthcare and so-called ‘femtech’ [8] has led to basic research in this space being overlooked. And unless we know how women’s bodies work at a molecular, cellular and physiological level in health and disease, technological innovations run the risk of being built on false foundations.

Breakthroughs in our basic understanding of the biology of Women’s Health are happening, just not at the pace we need to gain momentum. Over the past couple of years, our understanding of the molecular mechanisms of placental regulation has expanded [9,10], but we are a long way from being able to prevent pre-eclampsia and other placenta-related pregnancy complications. Similarly, we are starting to see progress in understanding how menopause and hormone therapy affect Alzheimer’s disease risk [11–13], but it will be a long time before this can be translated into clinical guidance or therapies, or until we understand how hormone-related therapies used by people of any gender affect future disease risk. Basic research has also revealed insights into the genetic basis for hyperemesis gravidarum (severe vomiting in pregnancy) [14], which will hopefully provide new leads for further research in this area. But more is needed. We need more funding for basic research into female physiology and better model systems to work with that capture the complexity of hormone dynamics over time. As Jennifer Garrison points out in her recent Perspective in the journal, we also need a more holistic view of female bodies that does not treat reproductive organs as separate to the health of the whole person.

Until we stop treating female physiology as too difficult to bother with, we will never build the underlying knowledge base that is needed to reform women’s healthcare. Yes, hormone cycles in females make the system more challenging to study, but scientists are supposed to relish a challenge. And surely, this is an area where truly groundbreaking discoveries can still be made. But to get there, funding bodies will need to create more opportunities for research into female physiology that will encourage existing labs to branch out and early career researchers to take on the challenge. Greater emphasis will need to be given to Women’s Health-related research in prestigious global events and conferences, to avoid it being relegated to specialist meetings. And publishers will need to value contributions in this area and give them a visible platform in broad-interest journals.

At *PLOS Biology*, we feel privileged to support research in this area. Over the past few years, we have featured thought-provoking Essays on the key research priorities to better understand sex differences in immunity and on how biological sex influences the immune system as it ages and what the repercussions are for healthcare, as well as research into the community dynamics of vaginal microbiota, ovarian aging, the role of taxanes in breast cancer recurrence, how sex and diet interact at the chromatin level, and how to adapt in vivo studies to include both sexes. We are proud to be able to offer a venue for publications that advance our knowledge of physiology for both sexes.

What affects the health of women affects us all and, ultimately, if we all pull together, this will become a challenge that can be overcome.
